# Multi‐institutional analysis of the prognostic significance of postoperative complications after curative resection for gastric cancer

**DOI:** 10.1002/cam4.2439

**Published:** 2019-07-29

**Authors:** Mitsuro Kanda, Seiji Ito, Yoshinari Mochizuki, Hitoshi Teramoto, Kiyoshi Ishigure, Toshifumi Murai, Takahiro Asada, Akiharu Ishiyama, Hidenobu Matsushita, Chie Tanaka, Daisuke Kobayashi, Michitaka Fujiwara, Kenta Murotani, Yasuhiro Kodera

**Affiliations:** ^1^ Department of Gastroenterological Surgery (Surgery II) Nagoya University Graduate School of Medicine Nagoya Japan; ^2^ Department of Gastroenterological Surgery Aichi Cancer Center Nagoya Japan; ^3^ Department of Surgery Komaki Municipal Hospital Komaki Japan; ^4^ Department of Surgery Yokkaichi Municipal Hospital Yokkaichi Japan; ^5^ Department of Surgery Konan Kosei Hospital Konan Japan; ^6^ Department of Surgery Ichinomiya Municipal Hospital Ichinomiya Japan; ^7^ Department of Surgery Gifu Prefectural Tajimi Hospital Tajimi Japan; ^8^ Department of Surgery Okazaki City Hospital Okazaki Japan; ^9^ Department of Surgery Tosei General Hospital Seto Japan; ^10^ Biostatistics Center, Graduate School of Medicine Kurume University Kurume Japan

**Keywords:** adjuvant chemotherapy, gastric cancer, postoperative complication, prognosis

## Abstract

**Background:**

Insufficient data are available on the prognostic significance of complications after resection of gastric cancer. Therefore, we aimed to assess this gap in our knowledge by studying patients with resectable gastric cancer.

**Methods:**

A multi‐institutional retrospective database comprising clinical information of 3575 patients who received resection of gastric cancer from 2010 to 2014 at nine institutions. Grades 2 or greater complications of the Clavien‐Dindo classification were judged as clinically relevant postoperative complications, and their associations with postoperative survival were assessed. We assessed the effect of complications on times of initiation and continuation of postoperative adjuvant chemotherapy by S‐1.

**Results:**

A total of 2954 patients were included in the analysis. Clinically relevant postoperative complications occurred in 664 (23%) patients. Patients’ recurrence‐free survival rate incrementally decreased as the grade of complications became greater. Patients with abdominal complications (eg, leakage of pancreatic fluids, intra‐abdominal abscess, and anastomotic leakage) and those with nonabdominal complications (eg, pneumonia) experienced worse recurrence‐free survival compared to those without complications. Patients who had complications were generally at greater risk of disease recurrence, except for those who underwent laparoscopic surgery and those with pathological stage I. Delayed initiation and shorter continuation of adjuvant S‐1 chemotherapy was experienced by patients with postoperative complications.

**Conclusions:**

Postoperative complications adversely affected the prognosis in patients with resectable gastric cancer.

## 1 INTRODUCTION

Gastrectomy with systematic lymphadenectomy remains the backbone of curative treatment of patients who were present with resectable gastric cancer.^1^, ^2^ Despite the recent advances in imaging, surgical devices, and perioperative management, morbidity rate after gastrectomy is reportedly 20%‐30%.^3^, ^4^ Incidence of postoperative complications invariably contributes to a longer hospitalization, increased medical costs, and diminished quality of life. Furthermore, evidence indicates postoperative complications significantly correlated to subsequent poor prognosis.^5^, ^6^ Unfortunately, there is controversy over the evidence supporting the prognostic significance of postoperative complications.^7^ For example, previous studies often suffer from small sample size, data from a single‐institution, limited information on adjuvant treatment, and a prolonged period of time to acquire data leading to time‐dependent transition of standard treatment.^8^, ^9^, ^10^ Moreover, little is known about the correlations between types and severity of complications with prognosis as well as how complications affect the quality of adjuvant treatment.

Here, we analyzed a multicenter dataset, acquired within a 5‐year interval, to assess the prognostic significance of postoperative complications according to the type and severity in patients with resectable gastric cancer. Moreover, we evaluated the influence of postoperative complications on subsequent adjuvant chemotherapy.

## 2 PATIENTS AND METHODS

### 2.1 Patients

**Figure 1:**
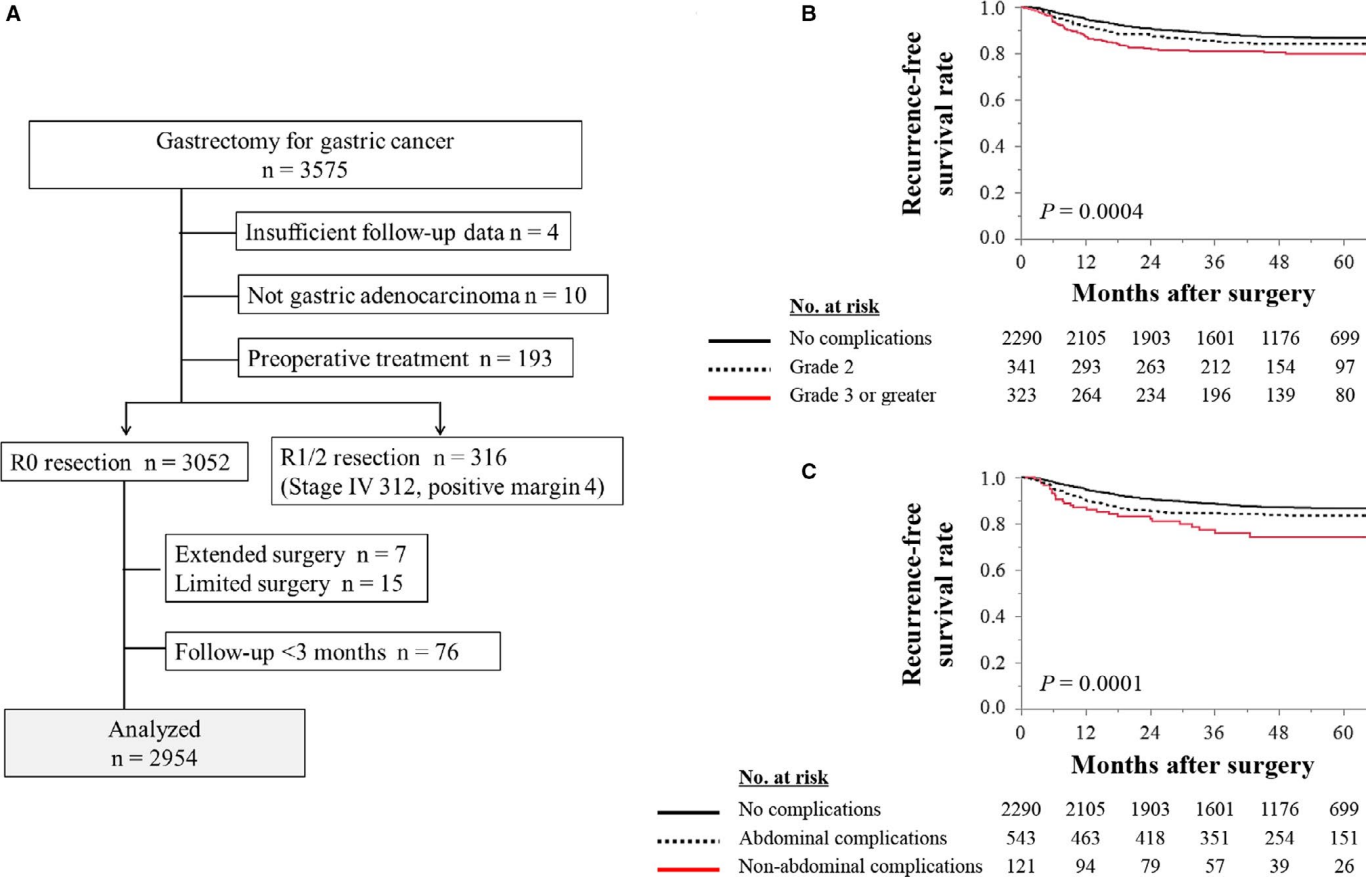
A, Study design. B, Recurrence‐free survival according to the severity of postoperative complications. C, Recurrence‐free survival according to types of postoperative complications

We analyzed an updated multi‐institutional retrospective database compiled by integrating clinical data from nine institutions and performed a retrospective review of the clinical data for 3575 patients who underwent gastrectomy for gastric cancer between January 2010 and December 2014.^11^, ^12^ The eligibility criteria of this study included histologically proven adenocarcinoma, no preoperative treatment, and no residual lesions after surgery (Figure 1A). The primary event of the present study was recurrence. Therefore, patients with <3 months follow‐up were excluded because early censored cases had little contribution to analysis of recurrence‐free survival times.

### 2.2 Surgery and postoperative management

We selected institutes that performed 50 or more surgical resection of gastric cancer a year to guarantee the quality of surgery. Partial gastrectomy includes distal gastrectomy, proximal gastrectomy, and pylorus‐preserving gastrectomy. A routine follow‐up after surgery consisted of laboratory tests including serum tumor markers every 3 months, contrasted CT scan every 6 months, and upper gastrointestinal endoscopy at 1, 3, and postoperative year in case patients have remnant stomach.^13^ For patients who were pathologically diagnosed as stage II or III, postoperative adjuvant S‐1 monotherapy or capecitabine plus oxaliplatin was recommended if tolerated.^14^ Treatment after recurrences was determined with consideration on the evidence available at the time of treatment and patient's condition.

### 2.3 Definition and categorization of postoperative complications

We used the Clavien‐Dindo classification for comprehensive evaluation of complications.^15^ Grades 2 or higher postoperative complications were regarded as clinically relevant, and their correlations with postoperative survival were assessed. Postoperative complications were categorized as abdominal (eg, surgical site infection, intra‐abdominal abscess, leakage of pancreatic fluids, anastomotic leakage, and bowel obstruction) or nonabdominal (eg, pneumonia, bacteremia, and urinary tract infection). To evaluate the prognostic significance of postoperative complications, subgroup analyses were conducted according to the type of gastrectomy, surgical approach, disease stage, and postoperative treatment. Moreover, we assessed the effect of complications on times of initiation and continuation of postoperative adjuvant chemotherapy by S‐1.

### 2.4 Statistical analysis

To compare patients with and without complications, the quantitative Mann‐Whitney and the qualitative Chi‐squared tests were employed. The Kaplan‐Meier method was used to estimate survival rates. To determine the hazard ratio (HR) for survival relative associated with each variable, we used the univariate Cox proportional hazards model. Multivariable regression analysis was performed to detect prognostic factors using the Cox proportional hazards model, and variables with *P* < .05 were entered into the final model. Statistical analysis was performed using JMP 13 software (SAS Institute Inc, Cary, NC, USA), and the presence of a statistically significant difference is denoted by *P* < .05.

## 3 RESULTS

### 3.1 Patients’ backgrounds

We included 2954 patients (mean age, 67.9 ± 10.4 years [±standard deviation], male‐to‐female ratio, 2113:841). Laparoscopic gastrectomy (n = 900, [30%]) or total gastrectomy was performed (n = 85, [29%]) (Figure 1A). Patients were pathologically diagnosed with stages IA (n = 1416), IB (n = 345), IIA (n = 291), IIB (n = 262), IIIA (n = 349), IIIB (n = 207), and IIIC (n = 84). Postoperative adjuvant chemotherapy was administered to 761 (26%) patients, and the median postoperative follow‐up was 51.1 months or until death (Table S1).

### 3.2 Incidence of postoperative complications

Grade 2 and ≥grade 3 postoperative complications were experienced by 341 (12%) and 323 (11%) patients, respectively (Table 1). Among abdominal complications, the prevalence of anastomotic leakage, intra‐abdominal abscess, and pancreatic fluid leakage was 4.0%, 3.0%, and 3.8%, respectively (≥grade 2). Among nonabdominal complications, postoperative pneumonia was experienced by 3.0% of patients (≥grade 2). A comparison of clinical characteristics between patients with postoperative complications ≥grade 2 (n = 664) and those who did not (n = 2290), revealed that the former were significantly older and had greater prevalence of cardiac and pulmonary comorbidities, larger tumor size, higher proportion of open surgery, total gastrectomy and D2 lymphadenectomy, longer operative time, greater blood loss, and more advanced disease stage (Table S2).

**Table 1:** Postoperative complications

	Grade 2	Grade 3 or greater
Overall	341 (11.5%)	323 (10.9%)
Abdominal complications		
Anastomotic leakage	32 (1.1%)	87 (2.9%)
Intra‐abdominal abscess	40 (1.4%)	49 (1.6%)
Pancreatic fluid leakage	21 (0.7%)	92 (3.1%)
Bowel obstruction	39 (1.3%)	26 (0.9%)
Stricture of anastomotic site	22 (0.7%)	30 (1.0%)
Delayed gastric emptying	38 (1.3%)	3 (0.1%)
Ascites fluids	12 (0.4%)	7 (0.2%)
Surgical site infection	13 (0.4%)	11 (0.4%)
Intra‐abdominal bleeding	8 (0.3%)	7 (0.2%)
Cholecystitis	10 (0.3%)	7 (0.2%)
Enteritis	12 (0.4%)	2 (0.1%)
Others	28 (1.0%)	12 (0.4%)
Nonabdominal complications		
Pneumonia	75 (2.5%)	16 (0.5%)
Bacteremia	14 (0.4%)	0
Urinary tract infection	6 (0.2%)	0
Delirium	5 (0.2%)	0
Cerebrovascular disease	3 (0.1%)	1 (0.03%)
Cardiac failure	1 (0.03%)	2 (0.07%)
Thrombosis	3 (0.1%)	0
Others	10 (0.3%)	3 (0.1%)

### 3.3 Recurrence‐free survival according to severity and types of postoperative complications

Patients were categorized according to the severity of postoperative complications as follows: none, grade 2, and ≥grade 3. Patients’ recurrence‐free survival rates were incrementally worse as the grade of complications increased (Figure 1B). Patients were next categorized according to the types of postoperative complications as follows: none, abdominal complications, and nonabdominal complications. Patients with abdominal or nonabdominal complications experienced shorter recurrence‐free survival compared with patients without complications (Figure 1C).

### 3.4 Prognostic impact of postoperative complications (≥grade 2)

**Figure 2:**
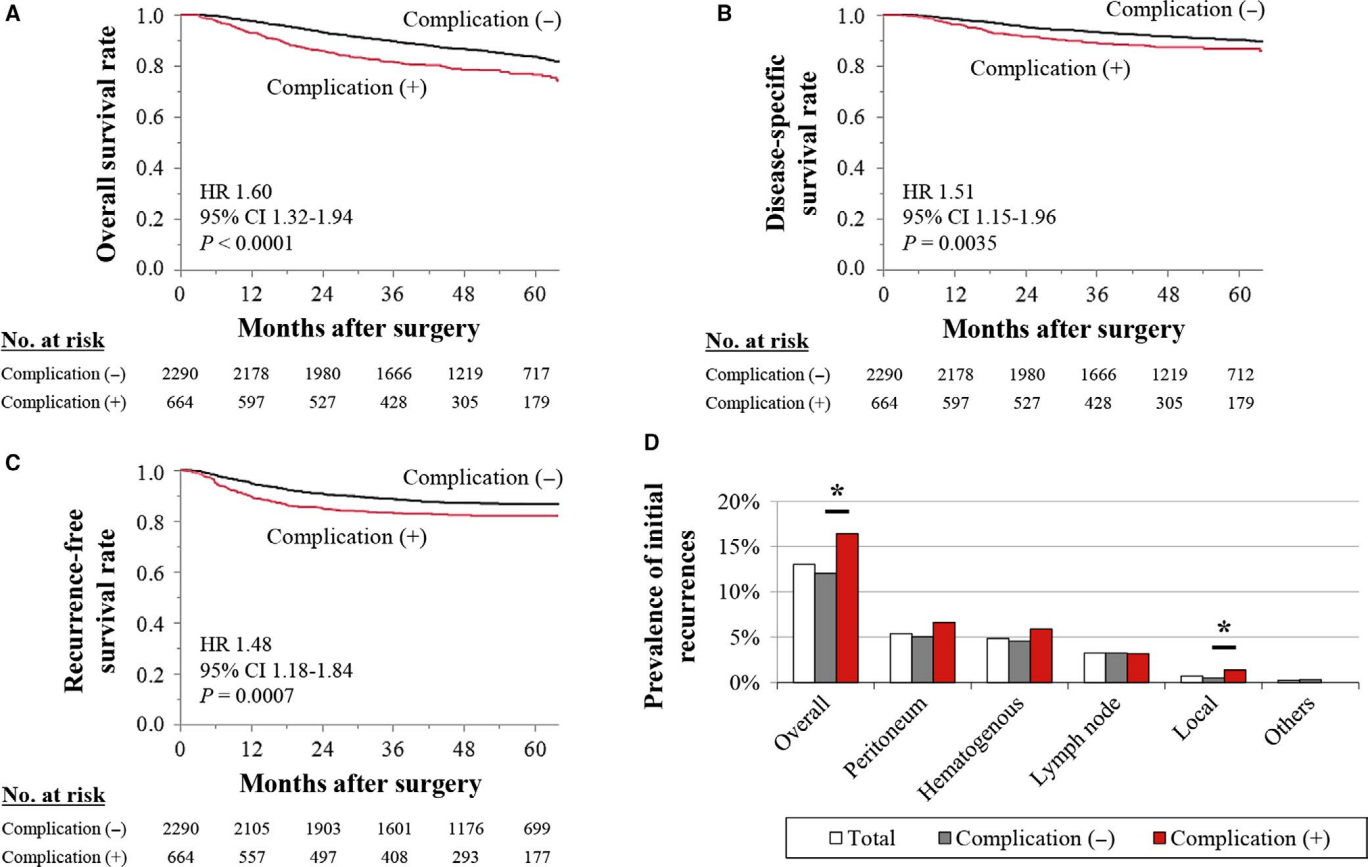
Comparison of prognoses between patients with and without postoperative complications of overall (A), disease‐specific (B), and recurrence‐free (C) survival. D, Prevalence of the sites of initial recurrences

**Figure 3:**
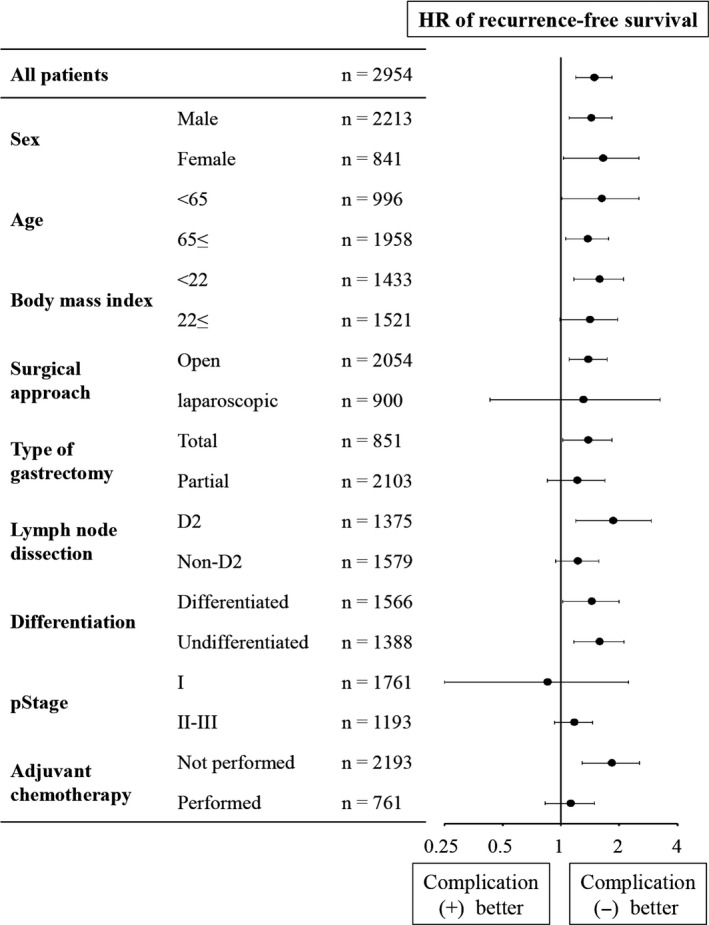
Significance of the association of postoperative complications with recurrence‐free survival

Overall survival was significantly shorter for patients with postoperative complications compared with those did not (HR 1.60, *P* < .0001) (Figure 2A). Disease‐specific and recurrence‐free survival were significantly shorter in patients with postoperative complications than in those without (HR, 1.51; *P* = .0035; disease‐specific survival [Figure 2B] and HR, 1.48; *P* = .0007; recurrence‐free survival [Figure 2C]). In multivariable analysis, postoperative complication was not identified as an independent prognostic factor (Table S3). Patients with postoperative complications had a greater overall recurrence rate compared with those without complications (16% vs 12%, respectively, *P* = .0041), reflecting significantly increased local recurrences and slightly higher frequencies of peritoneum and hematogenous recurrences (Figure 2D). To further assess the prognostic implications of postoperative complications experienced by patients with resectable gastric cancer, we conducted a subgroup analysis. A forest plot revealed that patients with postoperative complications were at greater risk of disease recurrence in most subgroups, except for the subgroups of laparoscopic surgery and pathological stage I (Figure 3). Recurrence‐free survival curves of patient subgroups according to pathological disease stages were shown in Figure S1.

### 3.5 Influence of postoperative complications on adjuvant treatment

**Figure 4:**
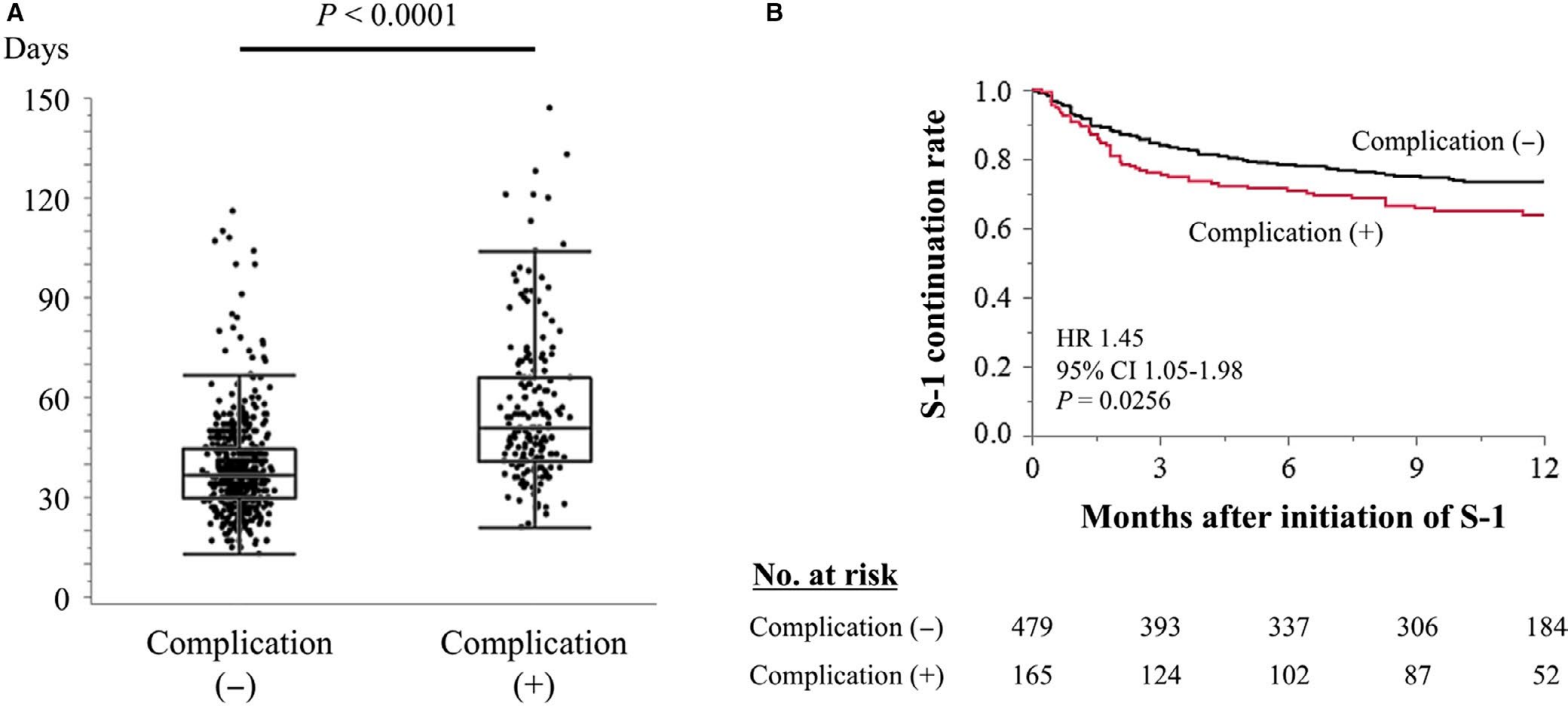
A, Comparison of the time between surgery and initiation of adjuvant S‐1 chemotherapy. B, Comparison of treatment continuation rates between patients with and without postoperative complications

We hypothesized that postoperative complications have undesirable effects on tolerability to the postoperative adjuvant chemotherapy, leading to a more unfavorable prognosis. Among 761 patients who received postoperative adjuvant chemotherapy, S‐1 monotherapy was administered to 689 (91%). The time between surgery and initiation of adjuvant S‐1 chemotherapy was significantly longer for patients with postoperative complications compared with those without (*P* < .0001) (Figure 4A). Moreover, patients with postoperative complications had a significantly lower continuation rate of postoperative S‐1 adjuvant therapy compared with those who did not (HR 1.45, *P* = .0256) (Figure 4B).

## 4 DISCUSSION

A multicenter dataset consisting of a large contemporary patient cohort, amassed over 5 years, was analyzed to determine whether postoperative complications adversely affected the prognosis after radical gastrectomy.^16^ We found that the incidence of postoperative complications was associated with worsened prognoses regardless of the severity and types of complications, in part, attributed to the undesirable effect on tolerability to adjuvant treatment by S‐1.

The adverse effect of complications after cancer surgery on survival has been reported in several solid malignancies, including colorectal and head and neck cancer as well as gastric cancer.^7^, ^17^, ^18^, ^19^ For example, Tokunaga et al analyzed 765 patients and found that grade 2 or higher intra‐abdominal infectious complications after radical resection of gastric cancer adversely affect survival.^5^ A propensity score matching was conducted by Fujiya et al to assess the prognostic impact of postoperative intra‐abdominal infectious complications graded 2 or higher, and they found that overall (HR 1.43) and recurrence‐free survival (HR 1.42) survival were significantly shortened in patients with complications.^9^ These results are consistent with those of the present study that focused exclusively on intra‐abdominal infectious complications.

The most frequently discussed hypothesis proposes that prolonged inflammation promotes the proliferation and metastasis of cancer cells, and suppresses immune responses.^20^, ^21^ It has been suggested that in mouse models, the adaptive immune system protects the host against carcinogenesis and eliminates cancer cells.^22^ Immunity mediated by cytotoxic T cells and natural killer cells can be compromised by surgical stress, particularly in the presence of postoperative complications.^22^, ^23^ Furthermore, excessive prostaglandin and catecholamine responses have adverse influence on immune system of the host, leading to progression of the disease and eventually shorter survival.^22^, ^24^ In the present study, HR of postoperative complications was higher for overall survival (1.60) rather than that for recurrence‐free survival (1.48). It was suggested that postoperative complications increased death from causes other than gastric cancer. Postoperative complications might impair patients’ activities of daily living and lead to a decline of muscle mass. Particularly for older patients, loss of muscle mass is associated with a decreased swallowing function and recurrent aspiration pneumonia.^25^, ^26^ Besides, reduced physical activity may promote the tendency of thrombus formation, leading to cerebrovascular and coronary artery events.

Limited information is available about whether the adverse effect of postoperative complications on prognosis depends on severity or types of complications.^10^ Our data demonstrated that incidence of complication was associated with worsened long‐term outcomes after resection of gastric cancer, which deteriorated proportionally to the elevation of the Clavien‐Dindo grade. This finding can be explained by strong suppression of tumor immunity caused by physical stress. Nevertheless, severe postoperative complications (grade 2) were linked to worse prognosis. After gastrectomy, complications include abdominal (eg, anastomotic leakage, leakage of pancreatic fluids, intra‐abdominal abscess, and bowel obstruction) and nonabdominal, including systemic reactions to surgical stress (eg, pneumonia, bacteremia, and thrombosis).^27^, ^28^ Little evidence is available on the prognostic significance of such postoperative complications. Here we conducted analyses focused on the prognosis of patients with abdominal or nonabdominal complications. We found that recurrence‐free survival times were shorter in both groups compared with patients without such complications. We therefore regarded all types of complications graded 2 or higher as events for further survival analysis. Consequently, we found that the overall survival of patients with postoperative complications was shorter. This may be attributed to the combined effects of tumor phenotypes and deteriorated quality of life. On the other hand, postoperative complication was not identified as an independent prognostic factor for recurrence‐free survival in the multivariable analysis. A possible explanation is that postoperative complication was closely associated with other strong prognostic factors including larger tumor size, total gastrectomy, and disease stages. A confounding with those factors would affect the results of postoperative complication in the multivariable analysis.

Our subgroup analysis contributed interesting findings. First, postoperative complications negatively influenced prognosis independent of sex, age, and physical condition. In contrast, postoperative complications had little prognostic influence on patients with stage I gastric cancer, possibly because a low risk of micrometastasis compensates impaired tumor immunity. Postoperative complications had little impact on prognosis of patient who received adjuvant chemotherapy. The most likely explanation for little prognostic impact of postoperative complications in patient who received adjuvant chemotherapy is that adjuvant chemotherapy compensated negative effects of complications.

The finding that the incidence of postoperative complications was associated with adverse prognosis regardless of the types of complications motivated us to consider reasons for their negative prognostic effects as well as decreased tumor immunity caused by inflammatory reactions.^24^, ^29^ We thus evaluated the influences of postoperative complications on the times of initiation and continuation of adjuvant S‐1 monotherapy. We found that delayed initiation and shorter continuation of adjuvant S‐1 chemotherapy was associated with patients with postoperative complications. To our knowledge, convincing data consistent with these findings have not been published subsequent to the implementation of standardization of postoperative adjuvant chemotherapy in Japan. We recently reported that delayed initiation of S‐1 is associated with worse prognosis.^30^ Moreover, the importance of the duration of administration is demonstrated by the OPAS‐1 trial.^31^ Our data suggest that postoperative complications confer a negative effect on prognosis through interference with adjuvant treatment. At the same time, it was considered that the dose of S‐1 within 1 year after surgery is of great importance, particularly in patients with postoperative complications.

A limitation of our study is its retrospective nature. Furthermore, insufficient immune‐nutritional data such as cytokine levels may have prevented us from acquiring a better understanding of the underlying mechanism of immunosuppression caused by postoperative complications. Detailed information on relative dose intensities, adverse events of postoperative adjuvant chemotherapy, and treatment after disease recurrences was unavailable this time.

In conclusion, a multi‐institutional dataset analysis indicates that postoperative complications had an adverse effect on prognosis after curative resection of gastric cancer.

## CONFLICT OF INTEREST

Nothing to declare.

## RESEARCH INVOLVING HUMAN PARTICIPANTS INFORMED CONSENT

The study protocol has been approved by the Institutional Review Board of all participating institutes. The ethical guidelines of the World Medical Association Declaration of Helsinki—Ethical Principles for Medical Research Involving Human Subjects were fully conformed when conducting the present study. A written informed consent for usage of data was granted from all patients before surgery.
