# Supplementary figures and images for: Multi‐institutional analysis of the prognostic significance of postoperative complications after curative resection for gastric cancer
Source: Cancer Med. 2019 Jul 29;8(11):5194–201. doi: 10.1002/cam4.2439 (PMC6718595; doi:10.1002/cam4.2439)

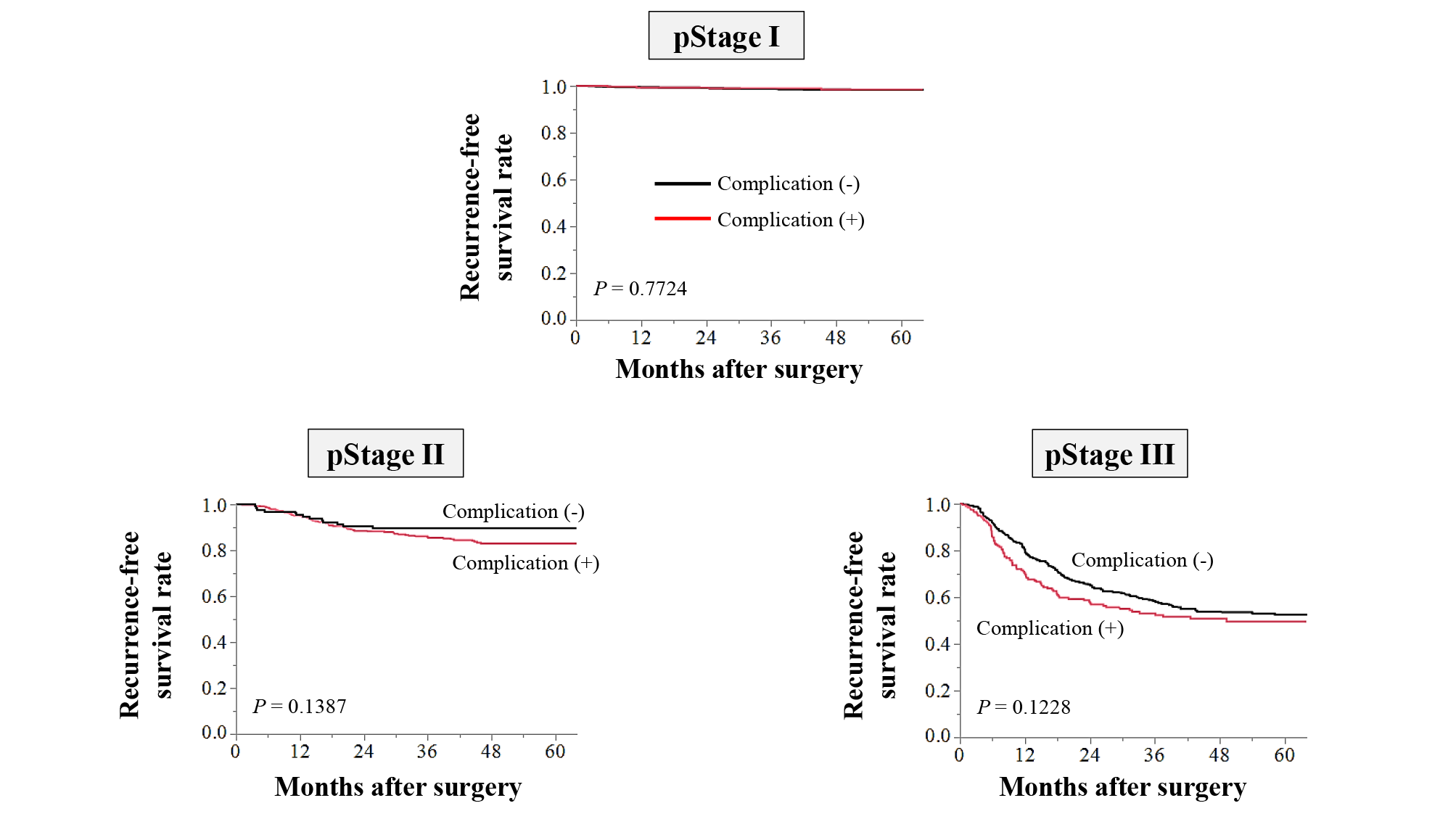

Supplement: Supplementary file 1 [file CAM4-8-5194-s001.tif]
