# Supplementary material for: Multi‐institutional analysis of the prognostic significance of postoperative complications after curative resection for gastric cancer
Source: Cancer Med. 2019 Jul 29;8(11):5194–201. doi: 10.1002/cam4.2439 (PMC6718595; doi:10.1002/cam4.2439)
Supplement: Supplementary file 2 [file CAM4-8-5194-s002.docx]

**Supplemental Table 1.** Patients’ demographics and perioperative clinical characteristics

| **Variables** |  |
| --- | --- |
| Age (years), mean ± SD | 67.9 ± 10.4 |
| Sex  Male  Female | 2113 (72%)  841 (28%) |
| Performance status  0  1  2 or higher | 2453 (83%)  393 (13%)  108 (4%) |
| Comorbidity  Cardiac disease  Pulmonary comorbidity  Cerebrovascular disease  Diabetes mellitus  Renal dysfunction | 343 (12%)  168 (6%)  181 (6%)  479 (16%)  74 (3%) |
| Preoperative body mass index, mean ± SD | 22.3 ± 3.2 |
| Tumor location  Entire  Upper third  Middle third  Lower third  Remnant | 53 (2%)  599 (21%)  1226 (42%)  1002 (34%)  39 (1%) |
| Tumor size (mm)  < 50  ≥ 50 | 2135 (72%)  819 (28%) |
| Approach  Open  Laparoscopic | 2054 (70%)  900 (30%) |
| Type of gastrectomy  Total gastrectomy  Partial gastrectomy | 851 (29%)  2103 (71%) |
| Dissected lymph nodes, mean ± SD | 33.0 ± 15.6 |
| Operative time (min), mean ± SD | 262 ± 74 |
| Estimated blood loss (ml), median (range) | 180 (0-6362) |
| Differentiation  Differentiated  Undifferentiated | 1563 (53%)  1382 (47%) |
| T factor  pT1  pT2  pT3  pT4 | 1611 (54%)  364 (12%)  459 (16%)  520 (18%) |
| N factor  pN0  pN1  pN2  pN3 | 1889 (64%)  440 (15%)  332 (11%)  293 (10%) |
| TNM stage  IA  IB  IIA  IIB  IIIA  IIIB  IIIC | 1416 (48%)  345 (11%)  291 (10%)  262 (9%)  349 (12%)  207 (7%)  84 (3%) |
| Postoperative adjuvant chemotherapy | 761 (26%) |
| Postoperative follow-up (months), median | 51.1 |

SD, standard deviation
