# Supplementary material for: Multi‐institutional analysis of the prognostic significance of postoperative complications after curative resection for gastric cancer
Source: Cancer Med. 2019 Jul 29;8(11):5194–201. doi: 10.1002/cam4.2439 (PMC6718595; doi:10.1002/cam4.2439)
Supplement: Supplementary file 3 [file CAM4-8-5194-s003.docx]

**Supplemental Table 2.** Comparison of clinical characteristics between patients with and without postoperative complications

| **Variables** | **Complication (+)** | **Complication (-)** | ***P*** |
| --- | --- | --- | --- |
| Age (years), mean ± SD | 69.6 ± 9.3 | 67.4 ± 10.6 | <0.0001 |
| Sex  Male  Female | 521 (78%)  143 (22%) | 2113 (70%)  841 (30%) | <0.0001 |
| Performance status  0  1  2 or higher | 528 (80%)  105 (16%)  31 (4%) | 1925 (84%)  288 (13%)  77 (3%) | 0.0241 |
| Comorbidity  Cardiac disease  Pulmonary comorbidity  Cerebrovascular disease  Diabetes mellitus  Renal dysfunction | 94 (14%)  49 (7%)  50 (8%)  108 (16%)  17 (3%) | 249 (11%)  119 (5%)  131 (6%)  371 (16%)  57 (2%) | 0.0227  0.0378  0.0943  0.9685  0.9179 |
| Preoperative body mass index, mean ± SD | 22.5 ± 3.4 | 22.2 ± 3.1 | 0.0298 |
| Tumor location  Entire  Upper third  Middle third  Lower third  Remnant | 18 (3%)  183 (28%)  229 (34%)  226 (34%)  8 (1%) | 35 (2%)  416 (18%)  1032 (45%)  776 (34%)  31 (1%) | <0.0001 |
| Tumor size (mm)  < 50  ≥ 50 | 419 (63%)  245 (37%) | 1716 (75%)  574 (25%) | <0.0001 |
| Approach  Open  Laparoscopic | 499 (75%)  165 (25%) | 1555 (68%)  735 (32%) | 0.0003 |
| Type of gastrectomy  Total gastrectomy  Partial gastrectomy | 258 (39%)  406 (61%) | 593 (26%)  1697 (74%) | <0.0001 |
| Lymph node dissection  D2  Non-D2 | 351 (53%)  313 (47%) | 1024 (45%)  1266 (55%) | 0.0002 |
| Dissected lymph nodes, mean ± SD | 33.7 ± 16.6 | 32.8 ± 15.3 | 0.3317 |
| Operative time (min), mean ± SD | 283 ± 79 | 256 ± 72 | <0.0001 |
| Estimated blood loss (ml), median (range) | 280 (0-6362) | 160 (0-3185) | <0.0001 |
| TNM stage  IA  IB  IIA  IIB  IIIA  IIIB  IIIC | 262 (40%)  68 (10%)  73 (11%)  62 (9%)  105 (16%)  69 (10%)  25 (4%) | 1154 (50%)  277 (12%)  218 (9%)  200 (9%)  244 (11%)  138 (6%)  59 (3%) | <0.0001 |

SD, standard deviation
