# Supplementary material for: Multi‐institutional analysis of the prognostic significance of postoperative complications after curative resection for gastric cancer
Source: Cancer Med. 2019 Jul 29;8(11):5194–201. doi: 10.1002/cam4.2439 (PMC6718595; doi:10.1002/cam4.2439)
Supplement: Supplementary file 4 [file CAM4-8-5194-s004.doc]

**Supplemental Table 3.** Prognostic factors of recurrence-free survival of 2954 patients with resectable gastric cancer

| **Variables** | | **Univariate** | | | **Multivariable** | | |
| --- | --- | --- | --- | --- | --- | --- | --- |
|  |  | **HR** | **95% CI** | ***P*** | **HR** | **95% CI** | ***P*** |
| Age (year) | ≥ 70 | 1.62 | 1.32 – 1.98 | <0.0001 | 1.53 | 1.22 – 1.92 | 0.0002 |
| Sex | Male | 1.07 | 0.85 – 1.33 | 0.5723 |  |  |  |
| Preoperative body mass index | ≥ 25 | 0.72 | 0.53 – 0.96 | 0.0253 | 1.15 | 0.83 – 1.59 | 0.3893 |
| Carcinoembryonic antigen | > 5 ng/ml | 2.24 | 1.75 – 2.87 | <0.0001 | 1.45 | 1.11 – 1.90 | 0.0061 |
| Carbohydrate antigen 19-9 | > 37 IU/ml | 3.10 | 2.39 – 4.01 | <0.0001 | 1.49 | 1.13 – 1.97 | 0.0047 |
| Tumor location | Lower third | 0.93 | 0.75 – 1.15 | 0.5095 |  |  |  |
| Tumor size | ≥ 50 mm | 4.21 | 3.43 – 5.15 | <0.0001 | 1.26 | 0.98 – 1.62 | 0.0680 |
| Multifocal lesion | Present | 1.47 | 0.93 – 2.33 | 0.1022 |  |  |  |
| Type of gastrectomy | Total | 2.79 | 2.29 – 3.41 | 0.0006 | 1.53 | 1.21 – 1.92 | 0.0003 |
| Postoperative complication | Grade II-VI | 1.48 | 1.19 – 1.85 | 0.0005 | 1.03 | 0.81 – 1.33 | 0.7884 |
|  | Grade III-VI | 1.65 | 1.25 – 2.17 | 0.0004 |  |  |  |
|  | Intra-abdominal infectious | 1.29 | 1.01 – 1.64 | 0.0424 |  |  |  |
| Tumor differentiation | Undifferentiated | 1.57 | 1.28 – 1.92 | <0.0001 | 1.15 | 0.90 – 1.48 | 0.2737 |
| Lymphatic involvement | Present | 10.4 | 7.16 – 15.1 | <0.0001 | 2.41 | 1.47 – 3.95 | 0.0005 |
| Vascular invasion | Present | 5.25 | 4.19 – 6.58 | <0.0001 | 1.36 | 1.04 – 1.79 | 0.0254 |
| Infiltrative growth type | Invasive | 3.23 | 2.63 – 3.96 | <0.0001 | 1.69 | 1.31 – 2.19 | <0.0001 |
| Pathological tumor depth | pT4 | 7.72 | 6.30 – 9.45 | <0.0001 | 2.10 | 1.62 – 2.72 | <0.0001 |
| Pathological lymph node metastasis | pN1-3 | 10.6 | 8.10 – 13.9 | <0.0001 | 3.48 | 2.47 – 4.91 | <0.0001 |

HR, hazard ratio; CI, confidence interval.
